# The Study of Prevalence and Distribution of Shape Anomalies of Teeth in Indian Population on the Basis of Age and Gender

**DOI:** 10.7759/cureus.28532

**Published:** 2022-08-29

**Authors:** Arvind Jain, Suruchi Sisodia, Kuldeep Singh Rana, Charvi Gupta, Iram Ansari, Poonam P Dholakia

**Affiliations:** 1 Conservative Dentistry and Endodontics, Government College of Dentistry, Indore, IND; 2 Conservative Dentistry and Endodontics, Modern Dental College and Research Centre, Indore, IND; 3 Conservative Dentistry and Endodontics, Pacific Dental College, Udaipur, IND; 4 Conservative Dentistry and Endodontics, Modern Dental College, Indore, IND; 5 Periodontology and Oral Implantology, Sai Kripa Dental Hospital, Jammu, IND

**Keywords:** gemination, fusion, cusp of carabelli, talon cusp, taurodontism, shape anomaly, dilaceration, dental anomaly

## Abstract

Introduction: Dental anomaly of shape leads to various clinical dental pathologies requiring the intervention of a dental specialist. Early diagnosis and intervention in earlier stages can restore a near-normal dentition and esthetics. So, the present study was undertaken to determine the prevailing dental anomalies of shape and its various subtypes in various age groups and gender variations.

Materials and methods: Retrospective dental casts and radiographs were evaluated in 3,000 cases consisting of an equal proportion of males and females. Only cases with an age range from 10 to 40 years were included in the study. Dental anomalies of shape were evaluated for the presence of Gemination, fusion, talon cusp, dens evaginates, the cusp of carabelli, dens invaginatus, taurodontism, and dilacerations and recorded.

Result: A total of 538 (17.9%) cases were found to have dental anomalies of shape which included 288 (19.2%) males and 250 (16.6%) females. Dilaceration was found to be the most common (9.2%) dental anomaly of shape followed successively by taurodontism (3.7%), talon cusp (2.23%), and the cusp of carabelli (1.4%). Most of the shape anomalies showed male predilection. A higher prevalence of most dental anomalies of shape was found in the younger age group of 10 to ≤25 years as it is not linked with age but still has got importance to know while the treatment is carried out in this age group.

Conclusion: It is quite imperative to have complete knowledge of trends and patterns of shape anomalies in India. It will guide dental practitioners to formulate a treatment plan on the basis of existing prevailing anomalies.

## Introduction

Developmental dental anomalies (DDA) of the primary and permanent dentition can lead to short-term and long-term complications which in turn lead to irreparable damage to the tooth [1]. The clinical effects of alteration in the shape of a tooth due to DDA are seen in many dental specialties especially restorative dentistry, pediatric dentistry, orthodontics, and oral surgery. Diagnosis at early stages with knowledge of prevalence and distribution in various age groups and gender allows for optimal management of cases and their treatment planning [2]. Thus, it helps dental clinicians to intervene at an appropriate time to prevent the development of complications.

The tooth formation results from reciprocal interactions between epithelial and mesenchymal cells of the first pharyngeal arch [1,2]. The genes involved in root anomalies are Sonic hedgehog (SHH), WNT signaling pathway, Bone morphogenetic protein (BMP), Transforming growth factor beta (TGF-B), and Nuclear Factor I-C(NFIC) because they are involved in the signaling pathway between Hertwig's epithelial root sheath (HERS) and mesenchyme [3]. Aberration of morphogenesis during the cap and bell stages is believed to result in tooth anomalies with abnormal tooth shapes. The process of epithelial folding determines the shape and number of cusps [4]. Theories regarding the formation of taurodontism are variable and the failure of invagination of the epithelial root sheath diaphragm sufficiently early at the proper horizontal level is most commonly attributed [5]. These changes and defects in HERS involve failure of the epithelial diaphragm to form a bridge prior to dentin deposition resulting in large pulp chambers [6].

Dental anomalies in shape may arise from complex interactions between genetic, epigenetic and environmental factors during the intricate and lengthy process of dental development. Occasionally, it may be due to chromosomal defects that may be X-linked also and therefore their prevalence may show gender variation. Ezoddini et al. [7] found age variation in the prevalence of Taurodontism, gemination, dens in dente, and talon cusp in their study among the Iran population. There is insufficient data on prevailing dental anomalies of shape and its subtypes in the Indian population on the basis of age and gender. Hence the present study was undertaken to determine the prevailing dental anomalies of shape and its various subtypes in various age groups and gender variation.

## Materials and methods

A retrospective cross-sectional observational study was conducted on the dental casts with their complete medical records and intraoral peri apical radiographs in the Government Dental College, Indore, India. Only cases with an age range from 10 to 40 years were included in the study. Records of cases belonging to India were taken for evaluation. A total of 3,000 cases including both males and females in equal proportion were included in the study. Intraoral examination findings, Dental casts, and radiographs were taken and reviewed after dividing them into two study groups. The first study group ranges from 10yrs to ≤25yrs of age and the second group from >25 to 40yrs assuming that dentition is supposedly complete by 25 years of age. Comprehensive clinical examination findings, dental cast, and radiographs were evaluated for the presence of Gemination, fusion, Cusp anomalies, taurodontism, and dilacerations. Third molars were not included for evaluation.

Inclusion criteria included the patients who were not diagnosed with any serious childhood illnesses and systemic syndromes. Patients with no history of previous orthodontic treatment were included in this study. Exclusion criteria excluded Patients of age < 10 years and > 40 years. Patients with syndromes that could cause developmental dental anomalies such as Down’s syndrome, cleidocranial dysostosis, ectodermal dysplasia, cleft lip and palate, tooth extracted due to caries, trauma, or for orthodontic reasons, large restorations preventing observation of crown morphology and incompletely formed roots were excluded.

Criteria for selection of various anomalies and their subtypes

Shape Anomaly

Fusion and Gemination Confirmed after thorough Dental cast and radiographically. Fusion (developmentally due to fusion of two adjacent tooth buds)- two separate canals or two separate roots in a fused crown was observed radiographically. The infusion number of teeth is less than usual [8]. Gemination (an attempt of a single tooth bud to divide)- Bifid crown with common root and common canal [8]. A tooth was considered to have gemination if it has an enlarged crown with a normal root and the tooth count was normal. A tooth was recorded fused if the tooth crown and root were enlarged and the tooth count revealed a missing tooth [9].

Cusp of Carabelli, Talon Cusp and Dens Evaginatus

The cusp of carabelli is defined as the prominent accessory cusp distinctly visible and located on the palatal surface of the mesiolingual cusp of a maxillary molar. Identified clinically and on inspection of dental casts and evaluated only on fully erupted teeth [10]. Teeth with minimally formed fifth cusps such as small, indented grooves, and depressed were not included in this study. Talon cusp is defined as a talon cusp is a well-delineated additional cusp that arises from the cingulum or cementoenamel junction of the anterior teeth in the maxilla or mandible affecting both deciduous and permanent dentition. It extends at least half the distance from the cementoenamel junction to the incisal edge [10] or must extend at least 1mm beyond the cementoenamel junction (CEJ) [11] identified clinically and on inspection of dental casts and evaluated only on fully erupted teeth. Dens evaginatus is defined as an accessory enamel cusp found on the occlusal tooth surface of mandibular premolars. Besides this Molars and incisors can also be affected and appears as small, rounded nodule between the buccal and lingual cusps [8,12]. Identified clinically and on inspection of dental casts and evaluated only on fully erupted teeth. Dens invaginatus is defined as a deep surface invagination of the crown or root that is lined by enamel. It was detected radiographically. Taurodontism is an enlargement of the body and pulp chamber of a multirooted tooth, with an apical displacement of the pulpal floor and bifurcation of the roots with no constriction at the level of the cemento-enamel junction. Feichtinger and Rosiwall [13]. In the taurodont tooth, the distance from the furcation of the root to the CEJ is greater than the occlusocervical distance. It was confirmed radiographically. Dilacerations refer to angulation, or a sharp bend or curve, in the root or crown of a formed tooth. It was identified radiographically using the same radiographs.

Statistical differences were analyzed using the binomial test comparing two proportions and Fisher’s exact test and the p-value was mentioned for the particular study with a statistical significance of p-value < 0.05.

## Results

Among 3,000 cases in our study including both males and females in equal proportion, 17.2% (n=517) of cases had at least a single dental anomaly of shape with a maximum of three dental anomalies of shape in 0.4% (n=12) of cases. A total of 538 (17.9%) cases were found to have developmental dental anomalies of shape which included 288 (19.2%) males and 250 (16.6%) females. Tables 1, 2 show the prevalence of age and gender distribution of various dental anomalies of shape.

**Table 1:** Prevalence of various shape anomalies of teeth according to gender *- Statistically significant n: number of patients included in the examination %: number of patients included in the examination

Dental anomalies	Male, n=1500	Female, n=1500	Total, n=3,000	P-value
Shape anomaly	%	%	%	
Gemination	0.03	0	0.03	1
Fusion	0.03	0.03	0.06	1
Talon Cusp	1.2	1.03	2.23	0.62
Cusp of Carabelli	0.93	0.47	1.4	0.04 *
Dens Evaginatus	0.73	0.53	1.26	0.41
Dens Invaginatus	0	0.03	0.03	1
Taurodontism	1.6	2.1	3.7	0.17
Dilaceration	5.06	4.13	9.2	0.08

**Table 2:** Prevalence and distribution of shape anomalies of teeth on the basis of age. *- Statistically significant n: number of patients included in the examination %: number of patients included in the examination

Dental anomalies	Age 10-≤25 years			Age >25-40 years			P-value
	Male n=750	Female n=750	Total n=1500	Male n=750	Female n=750	Total n=1500	
Shape anomaly	%	%	%	%	%	%	
Gemination	0.06	0	0.06	0	0	0	1
Fusion	0	0	0	0.06	0.06	0.13	0.49
Talon Cusp	2.2	1.73	3.93	0.2	0.33	0.53	0.00001 *
Cusp of Carabelli	1.6	0.8	2.4	0.26	0.13	0.4	0.00001 *
Dens Evaginatus	1.3	0.8	2.13	0.13	0.26	0.4	0 *
Dens Invaginatus	0	0.06	0.06	0	0	0	1
Taurodontism	1.9	1.93	3.8	1.33	2.26	3.6	0.84
Dilaceration	5.8	4.66	10.5	4.2	3.6	7.86	0.013 *
Total	12.9	10	22.9	6.26	6.66	12.93	0.00001 *

Dilacerations were the most common dental anomaly found in a total of 9.2% (n=276) of cases being common in both males 10.1% (n=152) and females 8.26% (n=124). Also, it was the commonest shape anomaly of the younger group range of 10 to ≤25 years (10.5%) as well as older age group range of >25-40 years 7.86%.

Taurodontism was found to be the second most common dental anomaly of shape with prevalence of 3.7% (n=111). Followed by this talon cusp (2.23%) and cusp of carabelli (1.4%) were more prevalent shape anomalies seen.

Prevalence of taurodontism found in males was 3.2% and in females was 4.2%. Beside this in both the study groups also, it was found to be the second most common anomaly (10-25 years [3.8%] and 25-40 years [3.6%]). Taurodontism was more prevalent in females while all other shape anomalies were predominantly seen in males. Fusion was more commonly seen in age group of >25-45 years while all other dental anomalies were more commonly seen in younger age group of 10 to ≤25 years.

## Discussion

The study of prevalence and distribution of developmental dental anomalies is crucial, so as to have data to analyze the present frequencies, patterns of various subtypes, changing trends, and etiology of various anomalies. The prevalence of developmental dental anomalies of the tooth though widely studied but the distribution of various subtypes along with the gender and age distribution is still lacking. The present study was therefore undertaken, specifically on shape anomaly to acquire and document the data of the distribution of its subtypes on the basis of gender and age group variation. Our results showed 17.9% of dental anomalies of shape. However, many previous studies of shape anomalies are inconsistent with our study due to variable attributes such as diagnostic criteria for identifying and classifying anomalies, and racial and genetic factors. Besides this different sample size, age groups studied with regional variation also exist, which might have influenced the prevalence of shape anomaly.

Fusion and gemination

Table 3 shows the comparison of our study with previous studies of shape anomaly of gemination and fusion.

**Table 3:** Comparative evaluation of gemination and fusion with previous studies T: total; M: male; F: female

Previous study and year	Age group included	Total Number of subjects	Gemination Prevalence (%)			Fusion prevalence (%)		
			T	M	F	T	M	F
Ardakani et al. 2007 [14]	All ages	480	2.1	0.83	1.25	0.2	0.2	0
Guttal et al. 2010 [15]	> 14 years	20,182	0.004	0.004	0	0.08	0.06	0.02
Shashirekha,Jena 2013 in maxillary lateral incisors [16]	15-30 years	1,062	0.28	0	0	0.18	0	0
Lochib et al. 2015 [17]	3-5 years	,1000	0.2	0	0	0.3	0	0
Saberi et al. 2016 [18]	> 16 years	1,172	0.09	0.09	0	0.09	0	0.09
Bandaru et al. 2019 [19]	3- 15 years	5,000	0	0	0	0.04	0.04	0
Present study	10-40 years	3,000	0.03	0.03	0	0.06	0.03	0.03

Our study showed the intermediate prevalence of gemination and fusion with single cases of gemination in males and one case each in both genders in case of fusion. Although previous studies on fusion anomaly showed higher male prevalence in some studies viz Guttal et al., Yassin (M-0.6% F-0.2%) [20] and Bandaru et al. while higher female incidence by Saberi et al. Gemination and fusion are altogether referred to the as double tooth. Our study showed a 0.1% overall prevalence of double teeth in the studied population of age group 10-40 years. In 2002, Knezevic et al.'s study on 3,517 plaster models in Croatian population found a double tooth prevalence of 0.2% [21]. Mukhopadhyay et al. [22] found a prevalence of 0.4% in 4-6yrs children in West Bengal while Kathariya et al. [23] showed the combined prevalence of 3.0% in Maharashtra population. The combined prevalence of fusion and gemination of tooth ranged from 0.084 to 3% in previous studies with our study being intermediate between them. On comparing age groups consisting of 1,500 subjects in each group, a single male case of gemination was found in the younger group of 10 to ≤25 years while two cases of fusion were detected in the older age range of >25-40 years. While the study by Ardakani et al. in 2007 [14] found 10 cases of gemination and one case of fusion in 480 subjects < 20yrs of age and no cases of gemination and fusion in >20 years age group in Iranian population.

Talon cusp

Our study showed an overall prevalence of 2.23% with 1.2% males and 1.03% females. Our study showed comparatively higher prevalence with male predilection. Difference might be due to difference in composition of population under study with regional variation. Age group comparison showed higher statistically significant difference in the prevalence of 10 to ≤25 years (3.93%) and >25-40 years (0.53%) with p-value of 0.00001. Ardakani et al. (Iran) in 2007 found 3 cases of talon cusp in 250 subjects in less than 20 years while no cases of talon cusp over 20 years age group. Gonçalves‑Filho et al. in Brazil found a prevalence of 3.09% in children <12 years and 0.95% prevalence in teenagers > 12 yrs and adults [24] and was also corelated same by Goutham et al. [25]. Our age group study also showed higher prevalence in younger age groups with decreased prevalence with advancing age. In 2017, Fekonja [26] found 3.4% prevalence of talon cusp higher than ours with female predilection.

Cusp of carabelli

Overall prevalence was found to be 1.45% with 0.93% males and 0.47% females. Prevalence of previous studies has a wider range from 0.3% - 59.5%. Variable incidence was due to differences in criteria of case selection, a number of subjects along with regional and ethnical differences. Major studies show male predilection favoring our study having male prevalence of 5.06% and female prevalence of 4.13%. In the present study Age group comparison shows 2.4% of cases in 10 to ≤25 yrs and 0.4% of cases in >25 to 40 years age group with statistically significant difference [19,27-29]. 

Dens evaginatus

In the present study, prevalence of dens evaginatus was found to be 1.26% with 0.73% males and 0.53% females showing male predilection. Guttal et al. [15] show male:female ratio of 9:1. The previous studies of Lin et al. [30] and Reichart et al. [31] found prevalence of 4.08% in Taiwanese students, 1.07% with 50 DE in 4677 Vietnamese and 1.01% in Thai population with male:female ratio being 1:1.83, respectively. Our study showed intermediate prevalence among various studies. Age group comparison showed 2.13% of cases in 10 to ≤25 yrs and 0.4% of cases in >25 to 40 years age group with statistically significant difference.

Dens invaginatus

In the present study prevalence of dens invaginatus (DI) was found to be 0.03% with a single case detected in a female in 3,000 subjects. Previous different studies show variable range of prevalence from 0.02% to 0.8% with greater or equal frequency in males when compared to females. Gonçalves‑Filho et al. in Brazil found a single female case with prevalence of 0.62% in children <12 years and two male cases with 0.63% prevalence in teenagers > 12yrs and adults. In present study a single female case was found in age group of 10-25 years similar to previous studies.

Taurodontism

Overall prevalence was found to be 3.7% with 1.6% males and 2.1% females with higher female preponderance in both the age groups of our study. Previous studies show quite wider range of prevalence from 0.31% to 27.19%. It was due to variable range of number of subjects, study design and regional variation. Present study shows higher prevalence in age group of 10-25 years (3.8%) as compare to 25-40 years age group having prevalence of (3.6%). Previous studies also show higher prevalence in younger age groups.

Dilaceration

Our study showed overall prevalence of 9.2% with 5.06% males and 4.13% females showing male preponderance. Previous study by Gutta et al. [15] shows very less prevalence of 0.4%. Prevalence of other previous study ranges from 14.01% to 16.48% with mostly male predominance. Our study showed higher incidence in younger group of 10 to ≤25 years (10.5%) as compared to >25-40 years having incidence of (7.86%). However previous study shows higher prevalence in adults with one study by Goncalves-Filho et al. showing statistically significant difference. It may be due to variable age group and subject selection with regional and ethnical variation.

The major limitation is the limited sample size and just no observation of the genetic link with the associated anamoly was checked. The study can incorporate on a large scale and various populations so that it can be generalized about the anamolies related to the teeth. 

## Conclusions

Dilaceration was the most common dental anomaly of shape followed successively by taurodontism, talon cusp, and the cusp of carabelli. Most of the shape anomalies showed male predilection. However, taurodontism's second most common shape anomaly showed female predominance. Age group comparison showed a higher prevalence of most dental anomalies of shape in the younger age group just to give us the prevalence during the treatment of this age group. While comparing with the present study, there was wide variation in the prevalence of shape anomalies across the world. Such variation was due to genetic and environmental factors which require further study so as to analyze the etiological factors and their association with the regional variation of shape anomaly of teeth.
